# Endophytes as Producers of Peptides: An Overview About the Recently Discovered Peptides from Endophytic Microbes

**DOI:** 10.1007/s13659-014-0038-y

**Published:** 2014-09-10

**Authors:** Muna Ali Abdalla, Josphat C. Matasyoh

**Affiliations:** 1 Department of Food Science and Technology, Faculty of Agriculture, University of Khartoum, 13314 Shambat, Khartoum North Sudan; 2 Department of Chemistry, Egerton University, P. O. Box 536, Egerton, Kenya; 3 Present Address: Institut für Chemie/FG Organische Chemie, Müller-Breslau-Str. 10, 10623 Berlin, Germany

**Keywords:** Peptides, Endophytes, NRPS, Bioactivities, Genetic engineering

## Abstract

An endophyte is a fungus or bacterium that lives within a plant in a symbiotic relationship. Extensive colonization of the plant tissue by endophytes creates a barrier effect, where they outcompete and prevent pathogenic organisms from taking hold. This happens by producing secondary metabolites that inhibit the growth of the competitors or pathogens. In this way they play a very important role in the plant defence mechanisms. The metabolites produced by these endophytes fall within a wide range of classes of compounds that include peptides which are the focus of this review. Peptides are increasingly being selected for drug development because they are specific for their targets and have a higher degree of interactions. There have been quite a number of endophytic peptides reported in the recent past indicating that endophytes can be used for the production of peptide based drugs. Molecular screening for NRPS, which shows peptide producing capability, has also shown that endophytes are potential producers of peptides. The presence of NRPS also offers the possibility of genetic modifications which may generate peptides with high pharmacological activities. This review, therefore, aims to show the current status of peptides isolated from endophytic bacteria and fungi in the recent decade. Endophytes as potential sources of peptides according to NRPS studies will also be discussed.

## Introduction

An endophyte which is predominantly a bacterium or fungus has an endosymbiotic relationship with the plant host [1, 2]. Endosymbiosis can be defined as a type of symbiosis in which one organism lives inside the other each benefiting from the relationship [3, 4]. Although endophytes were found in all studied plant species, the endophyte/host plant relationship is not yet well understood [5–7]. This may involve competition among endophytic species in the host tissue interposed by production of antifungal metabolites and detoxification of such inhibitors produced by endophytes [8]. Although mycorrhizal fungi colonize plant roots and reside into the rhizosphere, endophytes live entirely within plant tissues and may develop within roots, stems and leaves, sporulate at plant or host-tissue senescence [9–11].

### What Do Endophytes Do?

Endophytes can cooperate with their host plant by producing secondary metabolites that can protect the plant providing the ability to defend against predators, help their hosts to adapt in different stress conditions for survival [12–14]. It has been reported that the occurrence of a mutualistic endophyte works as a “biological trigger” to stimulate the stress response system more efficiently than nonmutualistic plants [15]. Endophytes encourage plant growth in different ways, such as production of siderophores e.g. enterobactin [16], and plant growth regulators such as indole-acetic acid [17], they can also enhance plant growth through phosphate solubilizing activity [18]. Moreover, endophytic bacteria supply essential vitamins to plants [19].

### Ethnobotanical Approach

One of the efficient methodologies used to find interesting endophytic strains is to take an ethnobotanical approach. In this case the knowledge of native people who have relied on plants as medicines for centuries must be followed [20]. Traditional herbal medicines in developing countries play an important role in improving the health status of the population and preventing endemic and acute diseases [21]. Also in developed countries traditional herbal medicine has attracted great interest, reinforced by the green movements and an increasing aversion to synthetic materials [22]. Traditional Chinese medicinal plants are the most famous example. They are sources of biologically active compounds, providing raw materials for the pharmaceutical industry for more than 5000 years.

It is interesting to note the story of the peptides munumbicins, which are isolated from snakevine *Kennedia nigriscans.* This plant was discovered several years ago by a tribal leader, Reggie Munumbie as a medicinal source in Aboriginal Australians culture to treat open, bleeding wounds to preclude sepsis. From this plant the endophytic *Streptomyces* NRRL 3052 generated a series of wide-spectrum peptides known as munumbicins. Recently, at least 39 different *Streptomyces* spp. were delivered from several snakevine plants collected in various places in the Northern Territory, Australia [23, 24]. These findings confirmed that the world’s rainforests are a novel source of endophytic streptomycetes.

### Isolation of Endophytes

Although vacuum or pressure extraction technique was successfully used to isolate endophytic bacteria from grapevine [25], and citrus trees [26], however, the method needs woody stems, because softer plant materials will collapse under vacuum. Another technique suggested the extraction of plant sap by using a Scholander pressure bomb [27]. The most known and standard isolation procedure is the surface sterilization followed by plating of small sterilized piece of plant material onto nutrient agar [28, 29]. Regarding the surface sterilization technique, the collected plant must be processed immediately after collection. The plant parts, which could be leaves, steams, seeds and roots, should be cut into small pieces, in order to facilitate both surface sterilization and the isolation [30]. Surface sterilization steps are normally performed to ensure the elimination of surface microorganisms. Surface sterilization of plant segments normally involves treating the plant material with a strong oxidant or general disinfectant shortly, followed by a sterile rinse to remove residual sterilant. Sodium hypochlorite (NaOCl), diluted in water to concentrations of 2–10 %, is the most known surface sterilant. The most commonly used wetting agent is ethanol (70–95 %); since it has limited antibiotic activity. At the end segments are rinsed in sterile water or 70–95 % ethanol after treatment for 1 min to remove the sterilant [11]. Sterilized segments are plated onto malt extract or potato dextrose agar and nutrient agar, which are commonly used for fungi and bacteria respectively. Colony-limiting agents and antibiotics also are often used for primary isolations. Since pure colonies of either fungus or bacterium are isolated, further characterization and taxonomical steps should be performed.

### Historical Background

**Fig. 1:**
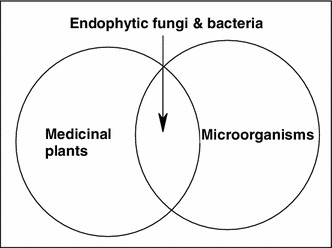
Metabolites of endophytes are overlapping between medicinal plants and microbes

As plants and microorganisms form close communities and as there is an increasing overlap between metabolites from microbes and plants; bacteria or fungi can produce secondary metabolites also inside the host plant. And indeed, some of the metabolites isolated from plant sources trace their origin back to endophytic microbes within the plants [31] (Fig. 1). The production of bioactive metabolites by endophytes might be connected to the independent development of these microorganisms. They may have combined genetic information from higher plants, which trigger them to adapt to the host and obtain defense functions such as protection from pathogens, insects, and grazing animals [32, 33]. Such cases, as that of gibberellin, where the biosynthetic mechanism of the same compound develops independently in plants and their microbial endophytes [34].

The most fascinating endophytic fungus is *Acremonium* sp. of the European yew (*Taxus baccata*). It yields a series of antifungal-anticancer peptides known as the leucinostatins; the most important of which is leucinostatin A, which demonstrated antifungal activity against the oomycetous plant-pathogenic fungus *Pythium ultimum* with an effective 1 day 50 % inhibitory concentration of <1 μmol. It also exihibits activity against certain human cancer cell lines, for instance, its IC_50_ value is 2.3 nM for breast cancer cell line BT-20 contrasted with 640 nM for a normal mammary cell line [35]. The lipopetide echinocandin A (from endophytic fungi *Cryptosporiopsis* sp. and *Pezicula* sp.) [36] and other echinocandins represent the first new antifungal class introduced for more than 25 years [37, 38]. They inhibit the synthesis of 1,3-β-D-glucan, an essential component of the fungal cell wall, and represents a valuable treatment option for fungal infections. They demonstrate potent in vitro and in vivo fungicidal activity against *Candida* species [39]. Cryptocandin (from endophytic fungus *Cryptosporiopsis quercina*) [40] is an antimycotic drug against multiple human pathogens including *C. albicans and Histoplasma capsulatum* (causal agent of the lung disease Histoplasmosis), in addition to *T. rubrum* and *T. mentagrophytes* [41]. Its most important activity is the inhibition of the growth of a number of phytopathogenic fungi including *Sclerotinia sclerotiorum*, the fungus that causes white mold disease and affects over 400 plants species, and *Botrytis cinerea*, a famous necrotic fungus that primarily affects grapes [42]. 

A number of lipopeptide antimycotics are produced by endophytic bacteria *Pseudomonas syringae* and related species, such as syringomycins A1, E and G [43] and pseudomycins, exhibit broad-spectrum of antifungal activity [44, 45]. Ecomycins are a novel family of peptide antimycotics, isolated in 1997 from *Pseudomonas viridiflava*, a plant-associated bacterium; they have significant bioactivities against a wide range of human and plant pathogenic fungi. The minimum inhibitory concentration values for ecomycin B are 4.0 μg mL^−1^ against *Cryptococcus neoformans and* and 31 μg mL^−1^ against *Candida albicans* [46]. *Pseudomonas viridiflava* is an endophytic bacterium and is associated with the leaves of lettuce (*Lactuca sativa*) and many grass species.

## Recently Discovered Endophytic Peptides

This section describes endophytic peptides isolated in the last decade. The number of the isolated endophytic peptides was limited compared to other groups of natural products, such as polyketides from endophytic origin. On the other hand we observed that there are no differences concerning peptide structures isolated from endophytic and non endophtyic microorganisms. The endophytic peptides have the same amino acids, which can be found in the non endophytic bacteria and fungi. That means the endophytes used the same biosynthetic machinery, which are used by the non endophytes to produce peptides.

Two cyclotetrapeptides designated as cyclo-(L-Val-L-Leu-L-Val-L-Leu) and cyclo-(L-Leu-L-Ala-L-Leu-L-Ala) were isolated from the endophytic fungus (No. 2221) isolated from *Castaniopsisfissa* [47, 48]. 

The chemical characterization of the endophytic fungus *Talaromyces wortmannii*, isolated from *Aloe vera*, obtained two new cyclic peptides, talaromins A and B. Their structures were established on the basis of extensive NMR spectroscopic and mass spectrometric analysis [49].

Lane and coworkers identified a number of genes encoding non-ribosomal peptide synthetases (NRPSs) in mutualistic grass endophyte *Epichloë festucae*, and putatively encoding a ferrichrome siderophore-synthesizing NRPS [50, 51]. A non-ribosomal peptide synthetase gene (sidN) was characterized. A novel extracellular siderophore was elucidated as epichloënin A and found to be the major product of the SidN enzyme complex [52, 53]. Additionally, epichloënin B was identified as a triglycine variant along with epichloëamide and ferriepichloënin A in guttation fluid from ryegrass (*Lolium perenne*) plants infected with wild-type *E. festucae* and also detected at trace levels in wild-type *E. festucae* fungal culture.

The nutritional iron sources for *Herbaspirillum seropedicae* were the first structurally described serobactin A, B and C siderophores produced by endophytic bacteria [54]. 

Siderophores are small high affinity chelating molecules with masses below 2000 Da secreted by microorganisms [55]. Iron is a requisite nutrient for the growth and proliferation of bacteria and fungi. The most important property of siderophores is their high affinity for the ferric ion [56, 57]. Therefore, their main role is to provide the cell with nutritional iron [58, 59]. The production of siderophores is widespread among bacteria and fungi and is found even in higher plants. The structures of siderophores can differ depending on the major Fe^3+^ ligands in bacteria and fungi, which can be catecholates, hydroxamic acids, and α-hydroxycarboxylic acids [60]. According to the biosynthetic pathways, siderophores are classified as non-ribosomal peptide synthetases (NRPS)-dependent or NRPS-independent [61].

### Endophytic Peptides Designated as Anticancer Compounds

More than 60 % of the anticancer drugs currently in clinical use are natural products or natural product derivatives [62, 63]. The first study of the endophytic microorganism *Bacillus amyloliquefaciens* sp. isolated from the medicinal plant *Ophiopogon japonicas* afforded the discovery of antitumor exopolysaccharides derived from the genus *Bacillus*. These findings provide a promising natural product source with high therapeutic value for antitumor activity against gastric carcinoma cell lines, thereby establishing the development of new anticancer agents from endophytic microbes [64].

The endophytic fungus strain (No. 2524) was growing on *Avicennia marina* (Forsk.) Vierh. seeds collected in a Hong Kong mangrove delivered two new cyclic pentapeptides, cyclo-(L-Phe-L-Leu1-L-Leu2-L-Leu3-L-Ile) and cyclo-(Phe-Val-Leu–Leu-Leu). Cyclo-(L-Phe-L-Leu1-L-Leu2-L-Leu3-L-Ile) demonstrated inhibitory activity against the human cancer cell line Bel-7402. Cellular viability was 67 % at a dose of 15 μg mL^−1^, whereas no dose-related effects were detected for dosages between 15 and 500 μg mL^−1^ [65, 66]. 

In the course of screening of endophytic fungi, two cyclic lipopeptides, fusaristatins A and B were isolated from rice cultures of a *Fusarium* sp. YG-45. Fusaristatin B exihibited a moderate inhibitory effect on topoisomerases I (IC_50_: 73 μM) and II (IC_50_: 98 μM) without cleavable complexes. Moreover, fusaristatins A and B demonstrated the growth-inhibitory activity toward lung cancer cells LU 65 with IC_50_ values of 23 and 7 μM, respectively [67]. 

Depsipeptides, 1962A and 1962B, were isolated from the fermentation broth of the mangrove endophytic fungus (No. 1962) isolated from an old leaf of *Kandelia candel* collected in Hong Kong. Their structures were established to be 1962A, cyclo-(D-Leu-Gly-L-Tyr-L-Val-Gly-S–O-Leu), and 1962B, cyclo-(D-Leu-Gly-L-Phe-L-Val-Gly-S–O-Leu), respectively. Both of these recently isolated cyclo-depsipeptides have one d-amino acid. In the MTT bioassay, 1962A showed weak activity against human breast cancer MCF-7 cells [68]. 

A cytotoxic pullularins E and F were characterized recently from the endophytic fungus *Bionectria ochroleuca* [69]. 

Cycloaspeptide A was isolated for the first time from the endophytic fungus *Penicillium janczewskii* K. M. Zalessky isolated from the phloem of the Chilean gymnosperm *Prumnopitys andina*. It demonstrated low cytotoxicity towards human lung fibroblasts with IC_50_ ≥ 1000 μM [70]. 

### Antibacterial Endophytic Peptides

The search for novel structures from microorganisms has increased in the last four decades [71]. While, there is an urgent necessity for new antibacterial compounds as well as treatment strategies, to conquer the increased difficulty in controlling bacterial infections and levels of antibiotic resistance of the pathogenic strains [72]. Currently, the antimicrobial membrane-active peptides produced by microorganisms have great interest and are important targets of intensive investigations globally [73].

Analysis of the transcriptomic PD library of the endophytic *Fusarium tricinctum* from a shrub *Rhododendron tomentosum* provided an antimicrobial peptide named Trtesin. The expression of Trtesin transcripts was ≥1000 fold higher in the mRNA library originating from PDB-grown fungi and demonstrating high antimicrobial activity. Trtesin was cloned, expressed, and purified in pET32 and it consisted of 52 amino acids with 6 cysteine molecules. The molecular weight of Trtesin is 6138.92 Da. An additional N-terminal sequencing was performed to confirm the intact peptide, as well as to check the correct amino acid sequence. The MIC of Trtesin was determined against several bacteria as 64 μg/mL. Moreover the peptide demonstrated a mild activity against *F. oxysporum* in agar diffusion assay, as a zone of inhibition of 10 mm at 100 μg of the peptide [74].

*Paenibacillus* sp. strain Aloe-11, a Gram-positive bacteria isolated from the root of *Aloe chinensis* in the southwest region of China. The strain exhibited fascinating antibiotic activity and intestine colonization ability. Several giant nonribosomal peptide synthetase (NRPS) genes were identified in the genome of *Paenibacillus* sp. strain Aloe-11, which are involved in the biosynthesis of antibiotics such as fusaricidin [75] and bacitracin [76] and other unknown peptides. It is important to mention that bacitracin antibiotic disrupts both gram positive and gram negative bacteria by interfering with cell wall and peptidoglycan synthesis.

Antibacterial cyclo-(Pro-Thr) and cyclo-(Pro-Tyr) were produced by the fermentation broth of endophytic fungus *Penicillium* sp. isolated from the mangrove plant *Acrostichum aureurm.* Both compounds demonstrated activity against *Staphylococcus aureus* and *Candida albicans* [77]. 

### Antifungal Endophytic Peptides

Epichlicin a novel cyclic peptide was obtained from the endophytic fungus *Epichloe typhina*, of the timothy plant (*Phleum pretense* L.). The amino acids were sequenced by NMR and mass spectrometry experiments. Enantiomers of 3-amino tetradecanoic acid, the amino acid of epichlicin, were synthesized as authentic standards. The stereochemistry of the amino acids was determined by means of an advanced Marfey method and chemical manipulation. Epichlicin exhibited inhibitory activity against the spore of the pathogenic fungus of the timothy plant *Cladosporium phlei*, at an IC_50_ value of 22 nM [78]. 

Five hybrid peptide-polyketides, curvularides A–E, were obtained from the endophytic fungus *Curvularia geniculata*, isolated from the limbs of *Catunaregam tomentosa*. Structure elucidation for curvularides A–E was performed by analysis of spectroscopic data and single-crystal X-ray crystallography. Curvularide B demonstrated antifungal activity against *C. albicans*, and it also exhibited synergistic activity with a fluconazole drug [79]. 

The cryptic role of endophytic fungi as sources of novel bioactive peptides was emphasized by Pagnozzi and her coworkers on *Trichoderma citrinoviride* investigations. It is an endophytic fungus of cork oak, which was selected previously for its antagonistic potential against various fungal pathogens involved in oak decline. The strain was cultivated and a mixture of polypeptide antibiotics (peptaibols) belonging to the paracelsin family was identified [80]. Purification and analyses of the peptide mixture afforded seven new amino acid sequences. The peptide mixture showed strong antifungal activity toward seven important forest tree pathogens, and it was highly toxic in an *Artemia salina* (brine shrimp) bioassay [81]. It is important to note that peptaibols and peptaibiotics are a class of linear peptides having a high alpha-aminoisobutyric acid (Aib) content and produced by filamentous fungi, especially by the members of the genus *Trichoderma*. These antibiotics are economically important for their anti-microbial and anti-cancer properties as well as ability to induce systemic resistance in plants against microbial violation [82, 83]. A peptide collutellin A exhibited antifungal activity against plant pathogenic fungi *Botrytis cinerea* and *Sclerotinia sclerotiorum* with a MIC of 3.6 μg mL^−1^ after 48 h [84].

### Endophytic Peptides are Immunosuppressive Drugs

Several non-ribosomal peptides received attention due to their pharmaceutical importance as antibiotics or immunosuppressive drugs [85, 86]. Reported literature about *Colletotrichum* species isolated from medicinal plants afforded an immunosuppressive novel peptide collutellin A along with wide range of biologically active natural metabolites including activity of cancer cell lines [84].

Two new cyclodepsipeptides called trichomides A and B, respectively, were isolated recently from the endophytic fungus *Trichotheciumroseum*. Trichomide A has immunosuppressive effect more selectively than cyclosporine A. It was found that trichomide A decreases the expression of Bcl-2, increases the expression of Bax, and has a small or negligible effect on the expressions of p-Akt, CD25, and CD69 [87]. 

It is interesting to note that regarding the recent idea of plant–microbe interactions, several mechanisms that control the endophytic immunomodulation of host plants could be much more than that of the translocation of the effector proteins [88]. The endophytic microbes are usually an excellent producer of bioactive secondary metabolites [89, 90]. This knowledge along with the findings of a cross-kingdom difference in fundamental immunities, strengthened collectively the hypothesis that endophytic fungi and bacteria may be developed to produce small molecules which have potent immunosuppressive activity to mammal cells [91, 92].

A potential novel immunosuppressive peptide collutellin A was isolated in 2008 from an endophytic fungus *Colletotrichum dematium* collected from a *Pteromischum* sp. growing in a tropical forest in Costa Rica. In a comparison study with cyclosporine [93], collutellin A inhibited CD4^+^ T cell activation of interleukin 2 (IL-2) production with an IC_50_ of 167.3 ± 0.38 nM, while cyclosporin A in the same test yielded a value of 61.8 nM. This indicated the immunosuppressive activity of collutellin A by the inhibition of IL-2 production with very low concentration. Moreover cyclosporin A at or above 8 μg mL^−1^ demonstrated high levels of cytotoxicity on human peripheral blood mononuclear cells, in contrast collutellin A or DMSO (carrier) alone exhibited no toxicity, after 24 and 48 h of culture. The molecular weight of collutellin A is 1127.7 Da, and its amino acid residues are Ile, Val, Ser, N-methyl-Val and beta-aminoisobutryic acid in nominal molar ratios of 3:2:1:1:1 respectively. Independent lines of evidence suggest that the peptide is cyclic and sequences of Val-Ile-Ser-Ile and Ile-Pro-Val were delivered by MS/MS as well as Edman degradation methods.

### Novel Endophytic Peptides Demonstrating Multiple Activities

Interestingly, between 2003 and 2005 several peptides isolated from endophytic streptomycetes. These types of peptides exhibited numerous kinds of bioactivities such as antibacterial, antifungal and antimalarial activities. From our point of view the missing structures of these peptides prevented a comparison, which can be established between endophytic bacteria and fungi related structures. Moreover, concerning the multiple activities of these peptides are they depending on the presence of some special amino acids? For example un natural amino acids such as beta-aminoisobutryic were emerged as very promising tools in medicinal chemistry. Unfortunately, the questions will remain till those structures publish.

In 2003 Strobel and coworkers isolated the peptides kakadumycins from an endophytic streptomycete (NRRL 30566) isolated from a fern-leaved grevillea (*Grevillea pteridifolia*) tree in the Northern Territory of Australia [94]. Kakadumycin A was the main product and it was structurally related to a quinoxaline antibiotic, echinomycin [95, 96]. Kakadumycin A displayed better bioactivity than echinomycin. It demonstrated antibacterial activity against Gram-positive bacteria, especially against *Bacillus anthracis* strains, the minimum inhibitory concentrations are 0.2–0.3 μg mL^−1^ and 1.0–1.2 μg mL^−1^ for kakadumycin A and echinomycin respectively. Both echinomycin and kakadumycin A exhibited antimalarial activity against *Plasmodium falciparum* with LD_50s_ in the range of 7–10 ng mL^−1^. In macromolecular synthesis assays both kakadumycin A and echinomycin displayed the same effects on the inhibition of RNA synthesis. 

Coronamycin is a complex of novel peptide antibiotics isolated by Strobel and coworkers in 2004. Coronamycin is delivered by a verticillate endophytic *Streptomyces* sp. from an epiphytic vine, *Monstera* sp., which was found in the Manu region of the upper Amazon of Peru [97]. It showed activity against *pythiaceous* fungi and a human pathogenic fungus *Cryptococcus neoformans*. It displayed activity against *Plasmodium falciparum*, with an IC_50_ of 9.0 ng mL^−1^. The cytotoxicity of coronamycin against a primary mammary epithelial cell line (HMEC) delivered an IC_50_ of 5–10 mg mL^−1^, whereas taxol yielded a value of 30–40 mg mL^−1^. To our knowledge the structures of coronamycins are still not yet published, their molecular weight are 1217.9 and 1203.8 Da and a search in the Dictionary of Chapmann and Hall [98] previously showed no similarity with all present peptides. The closest chemical relative of coronamycin could be a cyclic peptide polymyxin B1, produced by *Bacillus polymyxa*, which has a mass of 1203 Da, but contains leucine, but not tryrosine or methionine. Coronamycin peptides could be new class of antibiotics.

In 2005 the same group of Strobel and coworkers discovered two novel peptides munumbicins E-4 and E-5 from an endophytic *Streptomyces* NRRL 30562, which was originally isolated from *Kennedia nigriscans*, snakevine, in the Northern Territory of Australia [99]. The plant was used for centuries by Aboriginal peoples to treat open bleeding wounds to prevent sepsis. Previously, the same endophytic bacteria afforded munumbicins A and B [100]. Munumbicins E-4 and E-5 exhibited antibacterial activity against gram-positive and gram-negative bacteria and antifungal activity against the plant pathogenic fungus, *Pythium ultimum* at 5.0 mg mL^−1^. In addition to antimalarial activity against *Plasmodium falciparum* with IC_50_ values of 0.50 ± 0.08 and 0.87 ± 0.0.26 mg mL^−1^ for E-4 and E-5, respectively. It is important to mention that the exact structures of E-4 and E-5 have not been published. Both peptide antibiotics have identical molecular weight (1445.00) but different retention times on HPLC. They considered as chromophoric peptides whose structures are uniquely different from the actinomycins.

## Endophytes as Potential Producers of Peptides

Nonribosomal peptide synthetases (NRPSs) a large database of novel NRPS gene sequences are present in microbial genomes and metagenomes [101]. They are large multimodular biocatalysts that utilize complex regiospecific and stereospecific reactions to assemble structurally and functionally diverse peptides compared to the ribosomal system [102]. These peptides have important medicinal applications such as antibiotics, anticancer agents, immunosuppressants, enzyme inhibitors, siderophores, herbicides, antifungals, insecticides, and anthelmintics [103]. Normally, the catalytic domains of NRPS select, activate or modify the covalently tethered reaction intermediates to control the iterative chain elongation process and product release, which occur during the ribosome-independent peptide synthesis [104]. It is important to mention that one NRPS gene cluster was discovered bearing a 30-kb DNA fragment, containing four genes (*lchAA*, *lchAB*, *lchAC*, and *lchAD*) involved in the biosynthesis of surface-active lipopeptides, such as lichenysin [105, 106].

### Current Molecular Screenings for NRPS in Endophytes

Currently, molecular screening for (NRPSs) in endophytes is being performed to assess the peptide-producing capability of isolated fungi or bacteria, which are important natural product targets nowadays. Moreover, the presence of NRPS could offer further genetic modifications, which may generate novel genetically modified peptides in the future with high pharmacological importance. In parallel, anticancer as well as antimicrobial bioassays were obtained for the crude extracts to determine the most active strains. In this respect we summarized the recent data concerning NRPSs screening in endophytes. NRPSs of unknown function were targeted in the fungal endophytes (genera *Neotyphodium* and *Epichloë*) in addition to these some novel endophytic NRPS genes have been characterized such as NRPS5 using a degenerate PCR screen [107].

Examples of genetic screening for NRPS and biological activities of the endophytes were reported from Chinese herbs. Most of the NRPS screening was afforded by Chinese research groups, hence the Chinese flora is very rich with medicinal plants, and this can be a suitable opportunity to focus on endophytes isolation, bioactivities study and screening of the NRPSs. Although the PKSs were also screened in parallel, we preferred to concentrate on the NRPS as an indicator of the potentiality of the isolated endophytes to produce peptides. Endophytic Streptomycetes associated with pharmaceutical plants from the rainforest in Yunnan province, China, displayed remarkable antitumour and antimicrobial activities. Additionally high frequencies of positive PCR amplification were obtained for NRPS (61.0 %) biosynthetic systems [108]. *Camptotheca acuminata Decne* collected from Yunnan University afforded ninety endophytic actinomycetes. The results of 16S rRNA gene sequences confirmed that the isolates belonged to 10 genera and 6 families. Around 33.4 % of the endophytic actinomycete cultures demonstrated antimicrobial activity. The non-ribosomal peptide synthetase (NRPS) sequences were detected by PCR in 45.6 % of studied strains [109]. Tropical plants collected from several locations in Papua New Guinea and Mborokua Island, Solomon Islands afforded 123 endophytic actinomycetes. All isolates were characterized by 16S rRNA gene sequencing to deliver 17 different genera. Rare genera, such as *Sphaerisporangium* and *Planotetraspora* were detected; they have never been previously reported to be endophytic. About 60 % of the extracts demonstrated bioactivity or displayed LC–MS profiles with spectra indicative of secondary metabolites. The 29 nonproductive strains were further investigated by the detection of putative nonribosomal peptide synthetase (NRPS) genes and all were positive [110].

An endophytic actinomycetes strain LCB-0297 isolated from Yew Podocarpus (*Podocarpus macrophyllus*) was characterized primarily as a genus of Streptomyces. It exhibited strong antimicrobial and anticancer activities. PCR check-screening of its antibiotic biosynthesis genes afforded non-ribosomal polypeptide synthetase (NRPS) genes confirmed that its potentiality for antibiotic biosynthesis genes [111]. The ethnomedical plants, *Forsythia suspensa* and *Solanum torvum*, collected in Chengdu, China afforded 14 Strains of endophytic actinomycetes. Ten of the strains showed inhibition to HepG2 cancer cell line in varied degrees, accounting for 71 % of total isolates, 3 strains exhibited antibacterial activity and one showed acute cytotoxicity and wide-spectrum of antibacterial activities. Based on 16S rRNA gene partial sequences, one strain was identified to genus *Kribbella,* and the remaining 13 strains belonged to genus *Streptomyces*. PCR screening of biosynthesis genes afforded 5 strains possessing NRPS genes. Endophytic actinomycetes are known to be potential for producing prolific bioactive compounds [112]. Panxi plateau in South-west Sichuan in China with its unique geographical and climatological characteristics is a habitat to a great variety of medicinal plants. It was reported that 560 endophytic actinomycetes were isolated from 26 medicinal plant species in Panxi plateau. 60 isolates were selected for 16S rDNA-RFLP analysis and 14 representative strains were chosen for 16S rDNA sequencing. According to the phylogenetic analysis, seven isolates were *Streptomyces* sp., while the remainder belonged to genera *Micromonospora*, *Oerskovia*, *Nonomuraea*, *Promicromonospora* and *Rhodococcus*. Antimicrobial activity analysis combined with the results of amplifying genes coding for nonribosomal peptide synthetase (NRPS) showed that endophytic actinomycetes had broad-spectrum antimicrobial activity and potential natural product diversity [113]. The total DNA extracts of 30 traditional Chinese herbs, which were screened to study the potential of endophytes to produce bioactive peptides, by the presence of NRPS genes. Six bacterial NRPS and three fungal NRPS gene fragments were successful identified by the four PCR screens. Analysis of the detected endophyte gene fragments afforded consideration of the possible bioactivity of the peptides produced by endophytes in medicinal herbs [114]. Eighteen actinomycete isolates from 6 Stemona earthnut samples were screened for NRPS and biological activity. It was found that the isolates belonged to 4 genera, *Streptomyces*, *Pseudonocardia*, *Micromonospora* and *Methylobacterium*. The isolates also showed distinguished antibacterial activities among them 13 strains showed antimicrobial activity against *Staphylococcus aureus* and/or *Pseudomonas aeruginosa*. Seventeen isolates were positive for NRPS genes. It was reported based on the results of study that endophytic actinomycetes from Stemona, dominated by *Streptomyces* and *Micromonospora*, have good secondary metabolic potential including peptides and could act as a promising resource for bioactive metabolite discovery in the future [115]. A survey for endophytic fungi was carried out in 12 different regions of 7 provinces in China, delivered 2 *Epichloe* species and 4 *Neotyphodium* species. An improved method was used for genomic DNA of the slow-growing fungal endophytes. The DNA was used as template to detect the NRPS genes of the endophytes. The resulting sequences afforded a high sequence similarity with the NRPS gene [116]. Endophytes were isolated from eight different anticancer plants collected in China. A functional gene-based molecular screening strategy was used to target nonribosomal peptide synthetase (NRPS) in endophytes, it was found that the isolated endophytes are capable of producing a plethora of peptides. Moreover, all of the endophytic culture broth extracts exhibited antiproliferative effects in at least one test assay, cytotoxic, antibacterial or antifungal [117]. Endophytic actinobacteria were obtained from *Artemisia annua*. A round 228 isolates were represented at least 19 different genera of actinobacteria were characterized. Concerning the antimicrobial bioassay, many of the isolates demonstrated activity against plant pathogens. Screening for NRPS by high frequencies of PCR amplification was performed and it was available in 32.5 % of the tested isolates. The herbicidal activity indicated that 19 out of 117 samples of fermentation broths totally inhibited the germination of *Echinochloa crusgalli* [118]. The above mentioned NRPS screening in endophytes is summarized in Table 1.

**Table 1:** Recent NRPS screening studies in endophytes

No	Name of the endophyte	Area	Host	Year	Ref.
1	*Neotyphodium* & *Epichloë*	New Zealand	Ryegrass	2007	[107]
2	Streptomycetes	Yunnan province, China	Chinese pharmaceutical plants	2008	[108]
3	Actinomycetes	China	*Camptotheca acuminata Decne*	2010	[109]
4	Actinomycetes	New york	Tropical plants	2010	[110]
5	*Streptomyces*	China	Yew Podocarpus	2011	[111]
6	Actinomycetes	Chengdu, China	*Forsythia suspensa* & *Solanum torvum*	2011	[112]
7	Actinomycetes	Panxi plateau, China	Chinese medicinal plant	2011	[113]
8	Different species of bacteria and fungi	China	Traditional Chinese herbs	2012	[114]
9	Actinomycete	Xishuangbanna, Yunnan Province,	Stemona earthnut samples	2012	[115]
10	*Epichloë* and *Neotyphodium*	7 provinces, China	Traditional Chinese herbs	2012	[116]
11	Different species of bacteria and fungi	China	Anticancer plants	2012	[117]
12	Actinobacteria	China	*Artemisia annua*	2012	[118]

## Engineering of Peptides: The Future Potential Drugs

NRPS enzymes are capable of synthesizing many peptide derivatives using just one enzyme complex. The non-ribosomal peptides are linear, cyclic, or branched cyclic and can be modified by glycosylation, N-methylation, or acylation [119, 120]. Several antibiotic, anticancer and immunosuppressive agents have been synthesized [121]. The most famous classical example is the immunosuppressant cyclosporine and new insecticidal inniatin derivatives [122]. Generally, it was reported recently that rather than using combinatorial chemistry to synthesize natural products derivatives, their biosynthetic pathways can be investigated at the genetic level. The biosynthesis of most of these natural products is controlled by single gene clusters. Research groups characterize these clusters and employ genetic engineering to synthesize the native compounds and their derivatives. One of most important candidates are non ribosomal peptides [123, 124].

Looking to the current achievements in peptides engineering as a powerful tool, we can conclude that the production of new novel peptide derivatives with pharmaceutical applications could be generated in vitro and in vivo using the NRPS [122, 125]. It has been reported previously of novel analogs of fungal cyclooligomer depsipeptide synthetase, which were obtained by a variety of combinatorial biosynthetic methods, including precursor-directed biosynthesis, mutasynthesis, combinatorial mutasynthesis, and total biosynthesis [17]. Recently, seven new beauvericin derivatives synthesized using the nonribosomal peptide synthetase BbBEAS from the entomopathogenic fungus *Beauveria bassiana* were discovered. Chemical diversity was generated by in vitro chemoenzymatic and in vivo whole cell biocatalytic syntheses using either a *B. bassiana* mutant or an *E. coli* strain expressing the *bbBeas* gene [126].

Peptides are giving rise to a push in chemodiversity approaches, which could be a fascinating route to novel medicinally and agriculturally important therapeutic agents for management of human and plant health [127, 128]. With rise in cancer patients and metabolic diseases like diabetes, large pharmaceutical and biotechnological companies are actively investing in the development of newer peptides for various applications and are also opting for newer technologies for the synthesis of peptides. A direction in peptide generation has been assessed, since cancer chemotherapy is facing major challenges due to its inability to deliver the correct amount of drug directly. In addition, it affects the normal cells in the body.

**Fig. 2:**
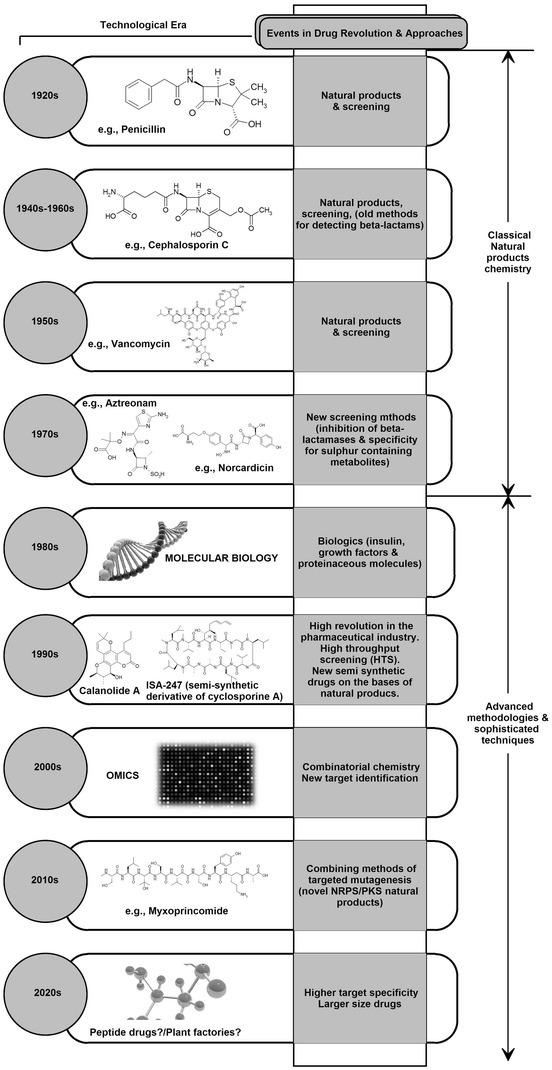
Important approaches in drug discovery

Looking to the longer term, we can speculate that methods of production and generation of peptide-based drugs will be more common in the future and considering the history of drug discovery, we can say that classical natural products and small compounds will be replaced by peptides generated by means of a combination of combinatorial biosynthesis, sophisticated genomic, proteomic and transcriptomic methodologies. The era of peptides and proteins as potential is already here before the expected 2020s (Fig. 2) [129–132].

## Conclusion

In conclusion, peptides will play a very important role in drug development as microbes increasingly show resistance to the current classes of antibiotics. The use of NRPS studies will also play a crucial role in the preparation of peptide based drugs because endophytes with the potential to produce peptides will be easily identified. Since endophytes from many parts of the world has not been studied, there is need to screen them using NRPS studies in order to create a database of these peptide producing microorganisms. Some of them may not produce peptides because their NRPS gene clusters are silent but a higher percentage will definitely be successful in peptide production. The research community should, therefore, focus their efforts on the biosynthetic mechanisms used by the non-ribosomal peptide synthetases of endophytes, which could lead to optimization of the production of peptides for biotechnological and pharmacological studies.
